# Self-reported environmental toxin exposure, awareness and preventive behavior among a university population in Kuwait: a cross-sectional study

**DOI:** 10.3389/fpubh.2026.1837341

**Published:** 2026-06-23

**Authors:** Suad M. Aladwani

**Affiliations:** Department of Environmental Sciences, College of Life Sciences, Kuwait University, Safat, Kuwait

**Keywords:** awareness, preventive behavior, self-reported environmental exposure, social demography, toxins

## Abstract

Exposure to environmental toxins is a critical concern that poses significant risks to human health and well-being. The detrimental effects of environmental degradation are linked to human health, as the consequences of water and air pollution have become apparent in the food we consume regularly. This study uses a cross-sectional quantitative survey design to evaluate self-reported environmental toxin exposure, awareness, and preventive behaviors among participants at Sabah Al-Salem University campus, Kuwait University. Data were collected across three domains (food and water, home and work environments, and lifestyle-related activities) from a total of 1,110 participants. All domains had acceptable to good internal consistency (Cronbach’s alpha = 0.71–0.79). A predetermined scoring framework was used to categorize self-reported exposure and knowledge levels, and Chi-square tests with Cramer’s V effect sizes were used to evaluate differences across sociodemographic Characteristics. To find independent determinants of moderate-to-high self-reported perceived exposure, binary logistic regression models were also built. The levels of overall self-reported perceived exposure were particularly high in the food and water domain, with 15.7% of individuals categorized as having high perceived exposure, in contrast to only 4.7 and 6.0% in the other domains. Participants showed the highest awareness of toxin perceived exposure from work and home environments (38.5%) compared to other domains. The study shows that a Kuwaiti university population had moderate levels of self-reported environmental toxin perceived exposure and awareness. In the food and water (OR = 1.66, *p* = 0.004) and lifestyle (OR = 1.50, *p* = 0.013) categories, female gender was independently linked to higher perceived exposure; whereas, in the home and work environment (OR = 0.68, *p* = 0.011), perceived exposure was lower. Residents of Al-Ahmadi (OR = 1.52), Hawali (OR = 1.58), and Mubarak Al-Kabeer (OR = 1.64) had higher odds than residents of Farwaniya, despite the fact that governorate of residency was substantially correlated with lifestyle perceived exposure. These results emphasize how urgently better training and educational initiatives aimed at younger university populations must be put into place.

## 1 Introduction

The advancements in industrialization and technological progress in our modern era have undoubtedly brought exceptional benefits to society. However, alongside these advancements, there has also been a concerning increase in toxic exposure. The term toxic exposure encompasses a broad range of substances, including heavy metals, pesticides, disinfectants, and hazardous chemicals, which can infiltrate our environment, bodies, and ultimately affect our well-being (1).

Toxic exposure can arise from a range of sources, whether natural or man-made, each carrying its own hazardous substance that can pollute the atmosphere (2). Industrial activities, power plants, and automobiles significantly contribute to atmospheric, water, and soil pollution, ultimately leading to the contamination of ecosystems (2). The use of pesticides and fertilizers in agriculture introduces harmful chemicals into our food supply, with traces of pesticides even detected in organic foods (3). Nowadays, the organic food market is experiencing growth in response to an increasing demand for organic products (including fruits, vegetables, grains, and animal products as opposed to non-organic options, primarily because they are free from chemical pesticides and fertilizers (4). Processed foods that have been subjected to blanching, cooking, canning, freezing, drying, dehydrating, mixing, packaging, or other methods contain preservatives and food additives (5). Bisphenol-A, a chemical used in the production of plastic containers, leaches into food when exposed to high temperatures and acidic conditions (pH) (6).

Lifestyle modifications have created a significant impact on toxin exposure among individuals. Sensitivity to personal care products such as lotions, moisturizers, shampoos, conditioners, deodorants, shaving creams and soaps; allergic to smoke, perfumes, incense, cleaning products, gasoline, silver fillings in the teeth; frequent coloring/straightening hair or regular visits to beauty salons; or regularly engaged with chemicals through hobbies which solvents, paints, stains, cleaners, or similar substances have notable effects. Household products, such as cleaning agents and paints, may emit toxins like volatile organic compounds (VOCs) within our living spaces, which have the potential to cause various adverse health effects (7). According to estimates, Americans utilize over 83,000 chemicals in their residences and workplaces, with only a small fraction having undergone safety evaluations (8). Exposure to smoke causes the inhalation of various toxic compounds such as carbon monoxide, hydrogen cyanide and formaldehyde, which can result in damage to the lungs and upper respiratory tissues (9). Exposure to paints can lead to heightened health risks because of inhaling a combination of pigments, solvents, fillers, binders, and other additives, which contribute to their chemical complexity. Personal care products can expose individuals to various chemical sources, including parabens, phthalates, and formaldehyde, which are of concern to human health (10). There have been investigations indicating a connection between EMF (telecommunication, electricity, appliances, medical equipment) and elevated occurrences of Leukemia, cancer, brain tumors, and other health complications (11).

The World Health Organization (WHO) stated that around 13 million deaths occur annually due to environmental exposure. Among these, more than 7 million deaths are directly associated with air pollution, particularly the presence of fine particulate matter (PM2.5) (12). Chronic exposure to heavy metals like arsenic, lead, and cadmium has been linked to severe DNA damage and various kidney and cardiovascular issues (13). Airborne pollutants enhance the prevalence of respiratory illnesses, cardiovascular problems, and serve as a potential for cancer development (1, 2). Moreover, exposure to endocrine-disrupting chemicals can lead to issues with reproduction and imbalances in hormone levels among humans (14). Persistent exposure to pesticides has also been shown to have detrimental effects on health, such as cancer and congenital disabilities (15). Soda and other processed beverages are sources of empty calories from added sugars, offering no nutritional benefits. Research has shown a strong link between consuming high amounts of sugar-sweetened drinks and a higher risk of developing obesity, diabetes, and heart disease (5).

A research study focused on examining the occupational exposure of research laboratory workers to hazardous chemical substances. The survey revealed that 54.4% of the workers felt highly exposed to chemical risks. Significant gaps in knowledge and insufficient preparedness in implementing safety measures to prevent and control risks associated with the use of chemical compounds in research laboratories were also noticed (16). The findings of a survey conducted among 1,000 European citizens indicated that a limited number of respondents were aware of the indirect negative consequences of plastic production on human health. This study further emphasized the importance of awareness, as it discovered a significant relationship between the information accessible to individuals and their choices about measures to reduce plastic consumption (17).

Numerous research studies have investigated the extent of the public’s understanding of ambient air pollution and its effects on health. In one study, authors surveyed 617 older adults to analyze their knowledge and preventive behaviors concerning PM2.5, emphasizing the need for health education among this demographic (18). Meanwhile, in another study, telephone interviews were conducted to assess the health literacy of individuals over 20 years old regarding ambient air pollution, revealing only moderate health literacy levels among individuals. Multiple linear regression models were used to identify the covariates significantly linked to their health knowledge. The analysis revealed that factors such as education, living situation, marital status, and geographic location were particularly linked to health literacy (19). The exposure of pregnant women to chlordecone, a pesticide used in banana farming, was examined through surveys and compared to the blood samples of the respondents. The attained results showed that surveys provided valid estimates of chlordecone exposure levels (20). A study involving pregnant women from various countries, conducted to assess environmental exposures at home and work, confirmed that most women did not encounter significant environmental hazards that could affect pregnancy outcomes according to the study’s criteria. However, some women reported concerns such as peeling paint, high residential density, the presence of pests, noise pollution, and safety issues in their homes (21). In another review study, more than 130 indoor air pollution studies were analyzed to understand the indoor air pollutants in GCC (Gulf Cooperation Council) countries. The study identified the following pollutants as the major indoor air pollutants in GCC, namely PM2.5, PM10, CO2, SO2, NO2, and total volatile organic compounds (TVOCs) (22). A survey-based cross-sectional study was performed with 827 female participants to evaluate their understanding, practices, and risk perceptions concerning foodborne illnesses. The results indicated a significant relationship between the respondents’ knowledge and practices and their employment status, Age, and educational attainment. The study highlighted a concerningly low level of knowledge among participants regarding “food cooking” (26.0%) and “risk of microbiological infection” (13.3%) (23) volatile organic compounds.

A holistic approach is essential for effectively tackling the consequences of toxic exposure, which entails implementing regulatory measures, raising public awareness, and fostering technological advancements. Public awareness campaigns are instrumental in educating individuals about the risks associated with toxic substances and encouraging sustainable choices in their daily routines. The objectives of this research study are: (1) to assess the level of self-reported perceived exposure and preventive awareness among teaching staff, non-teaching staff, and students at Sabah Al-Salem University campus; (2) to provide insights into preventive measures and awareness of environmental toxins across three domains; and (3) to identify associations between self-reported perceived exposure and awareness levels and selected socio-demographic characteristics.

## 2 Materials and methods

### 2.1 Study population

This cross-sectional questionnaire-based study assessed environmental toxin exposure and awareness among students, faculty members, and administrative staff at Sabah Al-Salem University campus, Kuwait University. Data collection was conducted between February and May 2024 using a self-administered online questionnaire distributed through Google Forms. A total of 1,110 eligible individuals were completed the questionnaire and were included in the final analysis. Participants were enrolled based on availability and willingness to participate. Individuals who did not complete the questionnaire were excluded. All exposure and awareness data are based entirely on participant self-report and do not reflect biomonitored, objectively measured, or clinically verified toxin levels.

Majority of the Students enrolled in the Sabah-Al-Salem university represents females (73%) (24). However, in the current study, a total of 1,110 participants which includes 876 women and 234 men representing 78.9% and 21.1%. Enrolled students, Faculty members and administrative staffs in the Sabah Al-Salem University Campus who only provided written informed consent were enrolled in the study. This study involved human participants and was approved by the Institutional Review Board of Kuwait University (Approval Number: KU-CLS-25-11-10). All procedures performed in studies involving human participants were in accordance with the ethical standards of the institutional research committee and with the 1964 Helsinki declaration. Written Informed consent was obtained from all individual participants of the study.

### 2.2 Survey design

A close-ended structured questionnaire was developed following detailed comparison of multiple studies examining occupational and non-occupational exposures to various toxins (25, 26). The questionnaire was pilot-tested on a representative sub-group; pilot responses were used to refine the instrument and were not included in the final analysis. The final survey consisted of 47 items organized into four domains: socio-demographic characteristics, food and water, home and work environment, and lifestyle-related activities.

Domain 1 contained five questions that covered the socio-demographic and professional information of the respondents, such as Age, gender, workplace (Graduate Studies College, Literature Colleges, Scientific Colleges, and Administrative Center), Occupation, and the governorate of residence (Farwaniya, Jahra, Hawali, Al-Asimah, Ahmadi, Mubaraq Al-Kabeer). The 2nd, 3rd, and 4th domains of the survey contained 15, 18, and 9 questions, respectively, aiming to explore the public’s understanding of various environmental toxins and to create awareness of different preventive measures. In domain 2 of the study, we explored the respondents’ knowledge concerning food and water, predominantly in relation to the consumption of processed foods and beverages. Additionally, respondents were investigated regarding preventive activities and awareness, such as avoiding toxin exposures from using plastic containers for storing hot food and beverages, and the benefits of choosing organically grown foods over non-organic ones. The 3rd and 4th domains assessed toxin exposures from home and work environments across various lifestyles adopted by the population, with a particular focus on the use of chemical-based personal care products. These domains also contained preventive and awareness information, including the emission of various toxic gasses from power plants, refineries, and other activities such as smoking, burning incense at home, and using chemical-based deodorants and perfumes.

### 2.3 Scoring

Responses were recorded using the options: yes, no, sometimes, in the past, and do not know. Only affirmative (“yes”) responses based on current observations were scored as 1 point. All other response categories scored 0. This binary scoring approach was intentionally selected to capture current, definitive perceived exposure or preventive practice. While this conservative approach may underestimate cumulative exposure burden for intermittent behaviours, it ensures that only current and definitive exposures are captured. Future studies should consider weighted scoring schemes that assign fractional values to responses such as “sometimes” to better account for infrequent but potentially meaningful exposures. Participants were classified into three levels: low (<=1/3 of items affirmed); moderate (>1/3 to <=2/3); high (>2/3). For every domain, Internal consistency was assessed using Cronbach’s alpha: Food and Water (15 items): alpha = 0.74; Home and Work Environment (18 items): alpha = 0.79; Lifestyle-Related Activities (9 items): alpha = 0.71. These values indicate acceptable to good internal consistency (27, 28).

### 2.4 Statistical analysis

**Figure 1:**
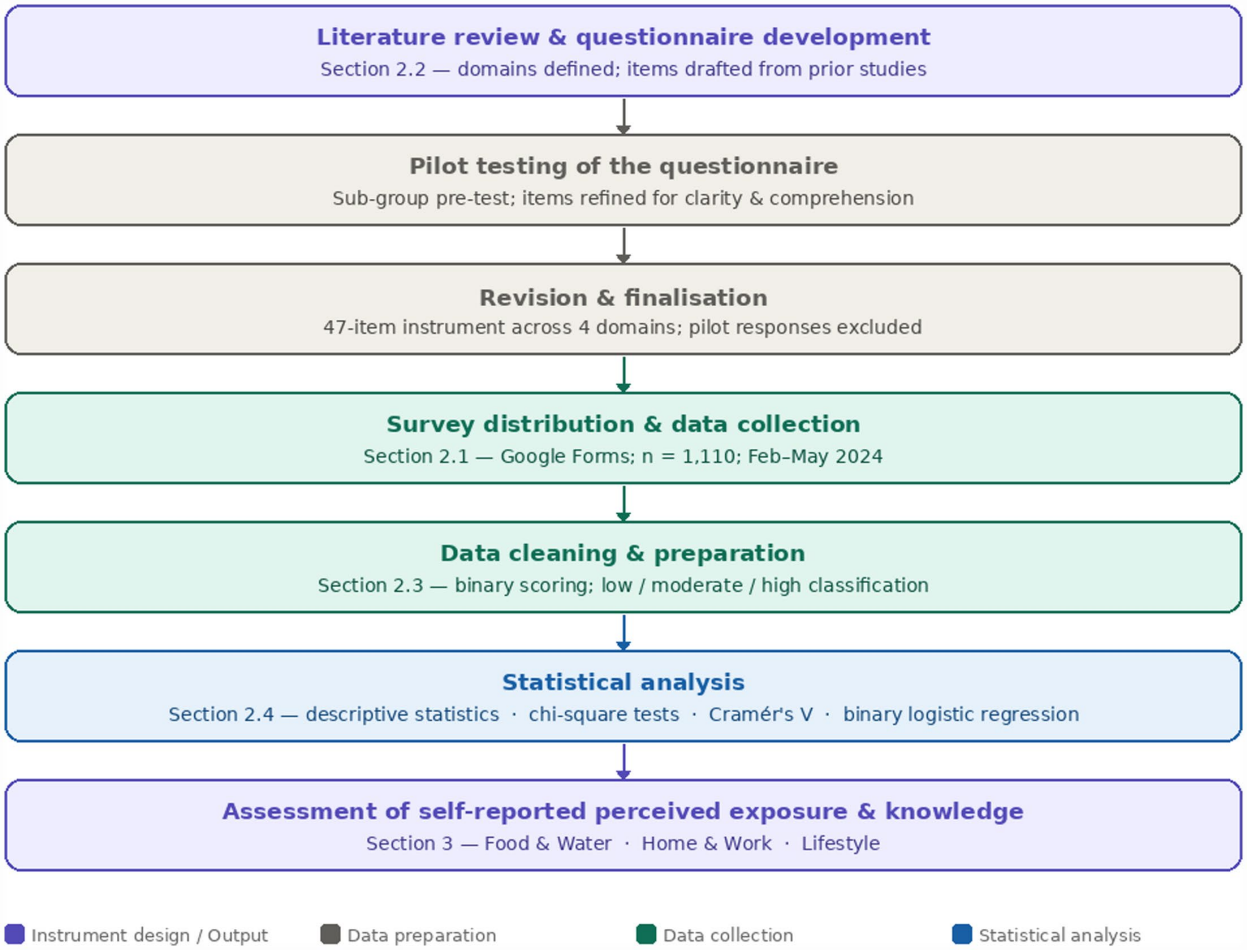
Study design and analytical workflow for assessing self-reported perceived exposure and knowledge levels.

Statistical analysis was performed using SPSS (version 29). Chi-square tests were conducted separately for two parallel outcome sets within each domain: (i) self-reported perceived exposure levels (Tables 1–3, Part A) and (ii) knowledge and preventive awareness levels (Tables 1–3, Part B), across all five socio-demographic variables. To measure the effect size for both outcome sets, Cramer’s V was computed alongside each Chi-square test. V values <=0.10 are negligible, 0.10–0.30 small, 0.30–0.50 moderate, and >0.50 large (29, 30). Binary logistic regression models were fitted separately for each domain to examine independent predictors of moderate-to-high self-reported perceived exposure (outcome), controlling simultaneously for age group, gender, governorate, occupation, and workplace. In Table 4, results are presented as odds ratios (ORs) with 95% confidence intervals (CIs). Statistical significance was set at *p* < =0.05. Given the cross-sectional design, all associations are descriptive and do not imply causality. A detailed preview of the study design is presented in Figure 1.

**Table 1:** Chi-square test results for self-reported perceived exposure (Part A) and knowledge (Part B)-food and water domain.

Characteristic	High *n* (%)	Moderate *n* (%)	Low *n* (%)	Chi^2^	*p*	V
A. Self-reported perceived exposure-food and water						
Age group						
17–24	125 (15.4)	514 (63.1)	175 (21.5)	10.98	0.359	0.070
25–30	21 (20.0)	61 (58.1)	23 (21.9)			
32–39	20 (14.5)	85 (61.6)	33 (23.9)			
40–47	9 (22.5)	22 (55.0)	9 (22.5)			
48–55	0 (0.0)	4 (50.0)	4 (50.0)			
>=55	0 (0.0)	5 (100.0)	0 (0.0)			
Gender						
Female	140 (15.9)	549 (62.7)	187 (21.3)	1.00	0.606	0.030
Male	35 (15.0)	142 (60.0)	57 (24.0)			
Governorate						
Al-Ahmadi	27 (13.5)	130 (65.0)	43 (21.5)	6.63	0.760	0.055
Al-Asimah	31 (15.8)	115 (58.7)	50 (25.5)			
Farwaniya	42 (14.0)	192 (63.8)	67 (22.3)			
Jahra	28 (16.6)	107 (63.3)	34 (20.1)			
Hawali	21 (17.2)	75 (61.5)	26 (21.3)			
Mubarak Al-Kabeer	26 (21.3)	72 (59.0)	24 (19.7)			
Occupation						
Administrative	8 (15.4)	31 (59.6)	13 (25.0)	2.41	0.661	0.033
Faculty	7 (22.6)	20 (64.5)	4 (12.9)			
Students	160 (15.6)	640 (62.3)	227 (22.1)			
Workplace						
Graduate studies	2 (15.4)	6 (46.2)	5 (38.5)	5.47	0.485	0.050
Literature colleges	120 (15.8)	464 (61.1)	175 (23.1)			
Admin centers	10 (19.6)	31 (60.8)	10 (19.6)			
Scientific colleges	43 (15.0)	190 (66.2)	54 (18.8)			
B. Knowledge and preventive awareness-food and water						
Age group						
17–24	225 (27.7)	396 (48.7)	193 (23.7)	34.80	<0.001	0.125
25–30	35 (33.3)	33 (31.4)	37 (35.2)			
32–39	60 (43.5)	48 (34.8)	30 (21.7)			
40–47	17 (42.5)	16 (40.0)	7 (17.5)			
48–55	3 (37.5)	4 (50.0)	1 (12.5)			
> = 55	4 (80.0)	1 (20.0)	0 (0.0)			
Gender						
Female	287 (32.8)	393 (44.9)	196 (22.4)	9.60	0.008	0.093
Male	57 (24.4)	105 (44.9)	72 (30.8)			
Governorate						
Al-Ahmadi	79 (39.5)	89 (44.5)	32 (16.0)	27.08	0.003	0.110
Al-Asimah	53 (27.0)	98 (50.0)	45 (23.0)			
Farwaniya	91 (30.2)	136 (45.1)	74 (24.6)			
Jahra	50 (29.6)	61 (36.0)	58 (34.3)			
Hawali	39 (32.0)	60 (49.1)	23 (18.9)			
Mubarak Al-Kabeer	32 (26.2)	54 (44.3)	36 (29.5)			
Occupation						
Administrative	17 (32.7)	28 (53.9)	7 (13.5)	8.50	0.075	0.062
Faculty	15 (48.4)	9 (29.0)	7 (22.6)			
Students	312 (30.4)	461 (44.9)	254 (24.7)			
Workplace						
Graduate studies	3 (23.0)	6 (46.2)	4 (30.8)	21.33	0.002	0.098
Literature colleges	217 (28.6)	332 (43.8)	210 (27.7)			
Admin centers	18 (35.3)	21 (41.2)	12 (23.5)			
Scientific colleges	106 (36.9)	139 (48.4)	42 (14.6)			

Chi^2^, Chi-square statistic. *V*, cramer’s V.

**Table 2:** Chi-square test results for self-reported perceived exposure (Part A) and knowledge (Part B)-home and work environment domain.

Characteristic	High *n* (%)	Moderate *n* (%)	Low *n* (%)	Chi^2^	p	V
A. Self-reported perceived exposure-home and work environment						
Age group						
17–24	35 (4.3)	321 (39.4)	458 (56.3)	14.14	0.167	0.080
25–30	8 (7.6)	43 (41.0)	54 (51.4)			
32–39	10 (7.3)	64 (46.4)	64 (46.4)			
40–47	0 (0.0)	22 (55.0)	18 (45.0)			
48–55	0 (0.0)	4 (50.0)	4 (50.0)			
> = 55	0 (0.0)	1 (20.0)	4 (80.0)			
Gender						
Female	39 (4.5)	343 (39.2)	494 (56.4)	7.89	0.019	0.084
Male	14 (6.0)	112 (47.9)	108 (46.2)			
Governorate						
Al-Ahmadi	15 (7.5)	87 (43.5)	98 (49.0)	8.71	0.559	0.063
Al-Asimah	6 (3.1)	79 (40.3)	111 (56.7)			
Farwaniya	16 (5.3)	121 (40.2)	164 (54.5)			
Jahra	6 (3.6)	68 (40.2)	95 (56.2)			
Hawali	3 (2.5)	49 (40.2)	70 (57.4)			
Mubarak Al-Kabeer	7 (5.7)	51 (41.8)	64 (52.5)			
Occupation						
Administrative	8 (15.4)	20 (38.5)	24 (46.2)	16.09	0.003	0.085
Faculty	1 (3.2)	17 (54.8)	13 (41.9)			
Students	44 (4.3)	418 (40.7)	565 (55.0)			
Workplace						
Graduate studies	0 (0.0)	7 (53.8)	6 (46.2)	5.79	0.447	0.051
Literature colleges	37 (4.9)	310 (40.8)	412 (54.3)			
Admin centers	5 (9.8)	23 (45.1)	23 (45.1)			
Scientific colleges	11 (3.8)	115 (40.1)	161 (56.1)			
B. Knowledge and preventive awareness-home and work environment						
Age group						
17–24	293 (36.0)	407 (50.0)	114 (14.0)	19.27	0.037	0.093
25–30	43 (41.0)	43 (41.0)	19 (18.0)			
32–39	62 (45.0)	60 (43.5)	16 (11.6)			
40–47	17 (42.5)	21 (52.5)	2 (5.0)			
48–55	4 (50.0)	4 (50.0)	0 (0.0)			
> = 55	5 (100.0)	0 (0.0)	0 (0.0)			
Gender						
Female	350 (40.0)	410 (46.8)	116 (13.2)	5.43	0.066	0.070
Male	74 (31.6)	125 (53.4)	35 (15.0)			
Governorate						
Al-Ahmadi	78 (39.0)	99 (49.5)	23 (11.5)	7.47	0.681	0.058
Al-Asimah	69 (35.2)	98 (50.0)	29 (14.8)			
Farwaniya	130 (43.2)	135 (44.9)	36 (12.0)			
Jahra	62 (36.7)	82 (48.5)	25 (14.8)			
Hawali	46 (37.7)	58 (47.5)	18 (14.8)			
Mubarak Al-Kabeer	39 (32.0)	63 (51.6)	20 (16.4)			
Occupation						
Administrative	27 (51.9)	20 (38.5)	5 (9.6)	8.46	0.076	0.062
Faculty	17 (54.8)	11 (35.5)	3 (9.7)			
Students	380 (37.0)	504 (49.1)	143 (13.9)			
Workplace						
Graduate studies	3 (23.1)	8 (61.5)	2 (15.4)	8.48	0.205	0.062
Literature colleges	285 (37.5)	368 (48.5)	106 (14.0)			
Admin centers	20 (39.2)	19 (37.3)	12 (23.5)			
Scientific colleges	116 (40.4)	140 (48.8)	31 (10.8)			

Chi^2^, chi-square statistic. *V*, cramer’s V.

**Table 3:** Chi-square test results for self-reported perceived exposure (Part A) and knowledge (Part B)-lifestyle activities domain.

Characteristic	High *n* (%)	Moderate *n* (%)	Low *n* (%)	Chi^2^	*p*	V
A. Self-reported perceived exposure-lifestyle activities						
Age group						
17–24	41 (5.0)	235 (28.9)	538 (66.1)	16.20	0.094	0.085
25–30	9 (8.6)	33 (31.4)	63 (60.0)			
32–39	11 (8.0)	50 (36.2)	77 (55.8)			
40–47	5 (12.5)	15 (37.5)	20 (50.0)			
48–55	1 (12.5)	1 (12.5)	6 (75.0)			
> = 55	0 (0.0)	3 (60.0)	2 (40.0)			
Gender						
Female	58 (6.6)	274 (31.3)	544 (62.1)	4.99	0.083	0.067
Male	9 (3.8)	63 (26.9)	162 (69.2)			
Governorate						
Al-Ahmadi	16 (8.0)	64 (32.0)	120 (60.0)	17.42	0.066	0.089
Al-Asimah	4 (2.0)	65 (33.2)	127 (64.8)			
Farwaniya	17 (5.6)	75 (24.9)	209 (69.4)			
Jahra	12 (7.1)	49 (29.0)	108 (63.9)			
Hawali	11 (9.0)	39 (32.0)	72 (59.0)			
Mubarak Al-Kabeer	7 (5.7)	45 (36.9)	70 (57.4)			
Occupation						
Administrative	6 (11.5)	13 (25.0)	33 (63.5)	11.33	0.023	0.071
Faculty	5 (16.1)	12 (38.7)	14 (45.2)			
Students	56 (5.5)	312 (30.4)	659 (64.2)			
Workplace						
Graduate studies	1 (7.7)	6 (46.2)	6 (46.2)	11.71	0.069	0.073
Literature colleges	44 (5.8)	234 (30.8)	481 (63.4)			
Admin centers	8 (15.7)	15 (29.4)	28 (54.9)			
Scientific colleges	14 (4.9)	82 (28.6)	191 (66.6)			
B. Knowledge and preventive awareness-lifestyle activities						
Age group						
17–24	63 (7.8)	435 (53.4)	316 (38.8)	12.87	0.231	0.076
25–30	6 (5.7)	57 (54.3)	42 (40.0)			
32–39	16 (11.6)	83 (60.1)	39 (28.3)			
40–47	5 (12.5)	21 (52.5)	14 (35.0)			
48–55	2 (25.0)	3 (37.5)	3 (37.5)			
> = 55	1 (20.0)	3 (60.0)	1 (20.0)			
Gender						
Female	78 (8.9)	495 (56.5)	303 (34.6)	14.03	0.001	0.112
Male	15 (6.4)	107 (45.7)	112 (47.9)			
Governorate						
Al-Ahmadi	24 (12.0)	117 (58.5)	59 (29.5)	13.26	0.210	0.077
Al-Asimah	14 (7.1)	104 (53.1)	78 (39.8)			
Farwaniya	24 (8.0)	163 (54.2)	114 (37.9)			
Jahra	15 (8.9)	82 (48.5)	72 (42.6)			
Hawali	11 (9.0)	67 (54.9)	44 (36.1)			
Mubarak Al-Kabeer	5 (4.1)	69 (56.6)	48 (39.3)			
Occupation						
Administrative	2 (3.8)	39 (75.0)	11 (21.2)	15.25	0.004	0.083
Faculty	6 (19.4)	17 (54.8)	8 (25.8)			
Students	85 (8.3)	546 (53.2)	396 (38.6)			
Workplace						
Graduate studies	1 (7.7)	9 (69.2)	3 (23.1)	4.65	0.590	0.046
Literature colleges	65 (8.6)	397 (52.3)	297 (39.1)			
Admin centers	5 (9.8)	29 (56.9)	17 (33.3)			
Scientific colleges	22 (7.7)	167 (58.2)	98 (34.1)			

Chi^2^, Chi-square statistic; *V*, Cramer’s V.

**Table 4:** Binary logistic regression: independent predictors of moderate-to-high self-reported perceived exposure by domain (*n* = 1,110).

Predictor	Food and water		Home and work		Lifestyle	
	OR (95% CI)	*p*	OR (95% CI)	*p*	OR (95% CI)	*p*
Gender (ref: male)						
Female	1.66 (1.18–2.33)	0.004	0.68 (0.50–0.92)	0.011	1.50 (1.09–2.08)	0.013
Age group (ref: (17−24))						
25–30	1.01 (0.60–1.70)	0.984	1.05 (0.68–1.61)	0.831	1.30 (0.84–2.02)	0.240
32–39	0.79 (0.51–1.24)	0.306	1.40 (0.95–2.05)	0.089	1.57 (1.06–2.32)	0.024
40–47	0.92 (0.40–2.12)	0.851	1.36 (0.70–2.65)	0.368	1.82 (0.93–3.57)	0.082
48–55	0.33 (0.07–1.56)	0.163	0.84 (0.20–3.57)	0.816	0.61 (0.11–3.36)	0.575
> = 55	0.54 (0.05–5.72)	0.609	0.24 (0.02–2.33)	0.217	1.77 (0.27–11.60)	0.554
Occupation (ref: students)						
Administrative	1.74 (0.78–3.90)	0.175	1.11 (0.60–2.06)	0.742	0.74 (0.39–1.42)	0.371
Faculty	3.48 (0.98–12.40)	0.054	1.62 (0.73–3.57)	0.235	1.81 (0.83–3.97)	0.136
Workplace (ref: literature colleges)						
Grad. Studies	1.72 (0.37–8.08)	0.489	1.15 (0.37–3.59)	0.807	2.08 (0.67–6.46)	0.208
Admin. centers	1.44 (0.66–3.13)	0.357	1.25 (0.69–2.29)	0.460	1.35 (0.74–2.48)	0.332
Scientific colleges	1.50 (1.04–2.15)	0.028	0.99 (0.75–1.31)	0.943	0.90 (0.67–1.22)	0.501
Governorate (ref: farwaniya)						
Al-Ahmadi	1.39 (0.89–2.19)	0.150	1.23 (0.85–1.77)	0.266	1.52 (1.03–2.22)	0.034
Al-Asimah	1.32 (0.84–2.07)	0.226	0.90 (0.62–1.30)	0.573	1.29 (0.87–1.91)	0.200
Jahra	1.12 (0.71–1.77)	0.615	0.92 (0.63–1.35)	0.678	1.34 (0.90–2.01)	0.154
Hawali	0.98 (0.59–1.63)	0.926	0.90 (0.59–1.40)	0.650	1.58 (1.01–2.48)	0.045
Mubarak Al-Kabeer	1.01 (0.61–1.68)	0.959	1.05 (0.68–1.61)	0.839	1.64 (1.05–2.55)	0.030

OR, odds ratio (moderate-to-high vs. low self-reported perceived exposure). All models adjusted simultaneously for all listed covariates (*n* = 1,110).

## 3 Results

A total of 1,110 eligible respondents completed the questionnaire. The age group 17–24 was most represented (73.3%). Female participants constituted 78.9% of the sample. The majority were from Literature Colleges (68.4%), and most resided in Farwaniya (27.1%). Detailed demographic characteristics are presented in Table 5.

**Table 5:** Demographic characteristics of study participants.

Characteristic	*n*	Percentage (%)
Gender		
Male	234	21.1
Female	876	78.9
Age group		
17–24	814	73.3
25–30	105	9.5
32–39	138	12.4
40–47	40	3.6
48–55	8	0.7
55 and above	5	0.5
Occupation		
Administrative staff	52	4.7
Faculty members	31	2.8
Students	1,027	92.5
Workplace (college)		
Graduate Studies	13	1.2
Literature Colleges	759	68.4
Administrative Centers	51	4.6
Scientific Colleges	287	25.9
Governorate of residence		
Farwaniya	301	27.1
Jahra	169	15.2
Hawali	122	11.0
Al-Asimah	196	17.7
Ahmadi	200	18.0
Mubarak Al-Kabeer	122	11.0

Table 1 presents Chi-square results for self-reported perceived exposure (Part A) and knowledge (Part B) regarding food and water toxins. Most participants attained moderate perceived exposure (62.2% overall) and moderate knowledge (44.8% overall). Specifically, the 17–24 age group showed 63.1% moderate perceived exposure. Age was not significantly associated with exposure (Chi^2^ = 10.98, *p* = 0.359, *V* = 0.070) but was significantly associated with knowledge (Chi^2^ = 34.80, *p* < 0.001, *V* = 0.125), with older participants showing greater awareness. Female participants demonstrated greater knowledge than males (Chi^2^ = 9.60, *p* = 0.008, *V* = 0.093). City of residence was significantly associated with knowledge (Chi^2^ = 27.08, *p* = 0.003, *V* = 0.110), with Al-Ahmadi residents showing the highest knowledge levels (39.5% high). Workplace was significantly associated with knowledge (Chi^2^ = 21.33, *p* = 0.002, *V* = 0.098). All effect sizes were small (*V* ≤ 0.125).

Table 2 presents results for the home and work environment domain. Most participants reported low self-reported perceived exposure (54.2% overall). Male respondents reported higher perceived exposure than female respondents (Chi^2^ = 7.89, *p* = 0.019, *V* = 0.084). Occupation was significantly associated with exposure (Chi^2^ = 16.09, *p* = 0.003, *V* = 0.085), with administrative staff showing the highest high-exposure proportion (15.4%). Age was significantly associated with knowledge (Chi^2^ = 19.27, *p* = 0.037, *V* = 0.093). All effect sizes were small (*V* ≤ 0.093).

Table 3 presents lifestyle domain results. Low self-reported perceived exposure was predominant (63.6% overall). Occupation was significantly associated with both exposure (Chi^2^ = 11.33, *p* = 0.023, *V* = 0.071) and knowledge (Chi^2^ = 15.25, *p* = 0.004, *V* = 0.083). Female participants showed significantly greater lifestyle knowledge than males (Chi^2^ = 14.03, *p* = 0.001, *V* = 0.112). All effect sizes were small (V ≤ 0.112).

Table 4 presents binary logistic regression results examining the independent predictors of moderate-to-high self-reported perceived exposure in each domain (*n* = 1,110), after simultaneously controlling for all socio-demographic covariates. Female gender was independently associated with higher odds of moderate-to-high perceived exposure in the Food and Water domain (OR = 1.66, 95% CI: 1.18–2.33, *p* = 0.004) and the Lifestyle domain (OR = 1.50, 95% CI: 1.09–2.08, *p* = 0.013). Conversely, female gender was associated with lower perceived exposure in the Home and Work domain (OR = 0.68, 95% CI: 0.50–0.92, *p* = 0.011), indicating that males show higher home and work perceived exposure. Scientific Colleges were associated with higher perceived exposure to Food and Water compared to Literature Colleges (OR = 1.50, 95% CI: 1.04–2.15, *p* = 0.028). In the Lifestyle domain, residents of Al-Ahmadi (OR = 1.52, *p* = 0.034), Hawali (OR = 1.58, *p* = 0.045), and Mubarak Al-Kabeer (OR = 1.64, *p* = 0.030) had significantly higher perceived exposure than Farwaniya residents. The age group 32–39 was also associated with higher perceived Lifestyle exposure compared to 17-24-year-olds (OR = 1.57, *p* = 0.024). Occupation, age, and governorate (for Food and Water and Home and Work domains) were not significant independent predictors after adjustment.

**Figure 2:**
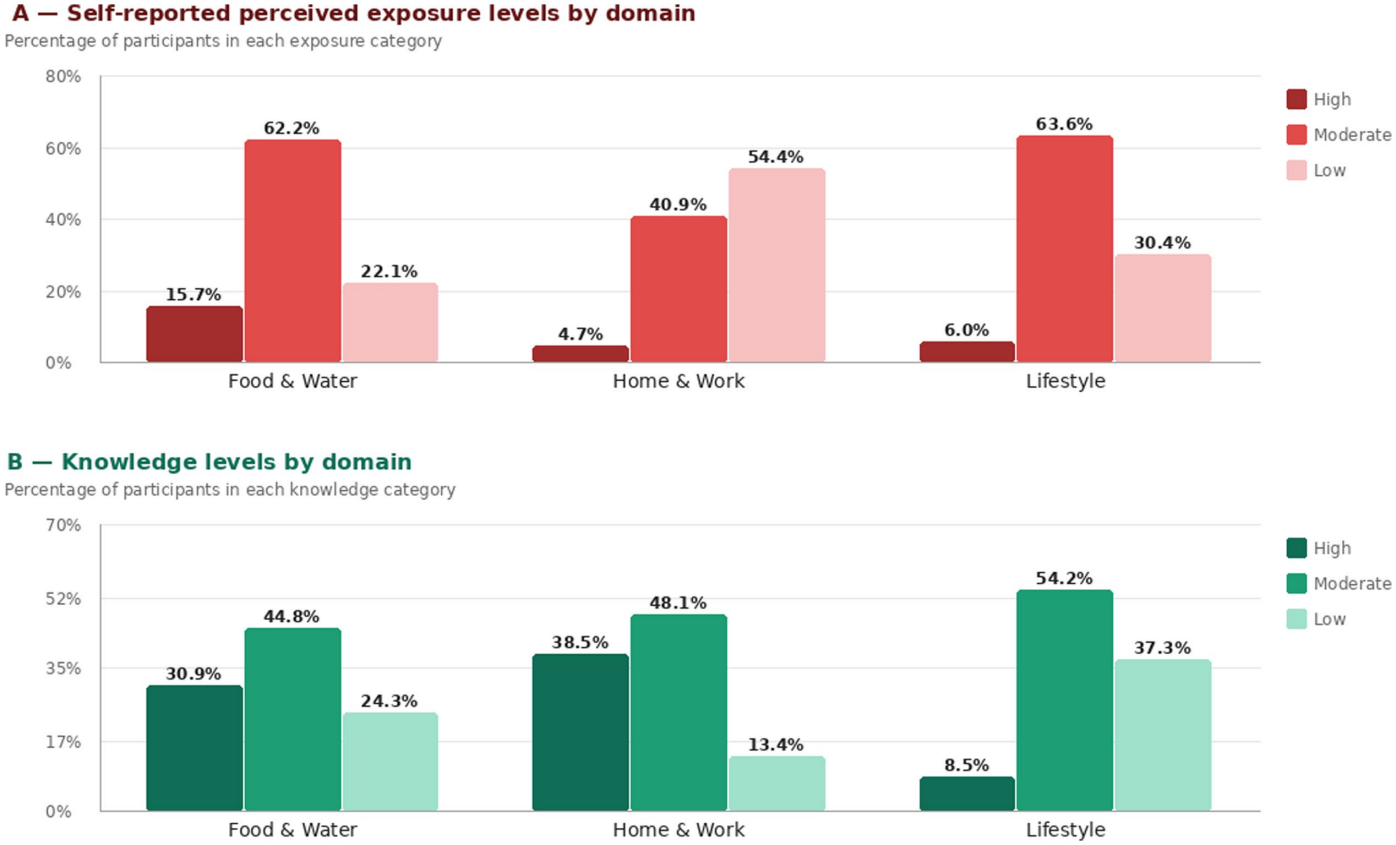
Distribution of overall self-reported perceived exposure **(A)** and knowledge levels **(B)** among participants across three domains. Percentage values are labelled on each bar. Moderate perceived exposure was the most prevalent category across all domains (40.9–63.6%). High perceived exposure ranged from 4.7% (Home and Work) to 15.7% (Food and Water). Knowledge was predominantly moderate across all domains (44.8–54.2%).

Figure 2 provides a summary of overall self-reported perceived exposure and knowledge distributions across all three domains. Moderate perceived exposure was the most prevalent category: Food/Water 62.2%, Home/Work 40.9%, and Lifestyle 63.6%. High perceived exposure remained consistently low (4.7–15.7%). Moderate knowledge was most frequent: Food/Water 44.8%, Home/Work 48.1%, and Lifestyle 54.2%.

## 4 Discussion

The study aimed to provide thorough statistical insights into self-reported perceived exposure levels and knowledge of preventive measures against toxins, categorized across three domains: demographics and environments in Kuwait. With age as a fundamental demographic variable, no statistically significant difference (*p* > 0.05) in self-reported perceived toxin exposure was observed across all three exposure domains. This was a descriptive trend rather than a definitive association (Chi^2^ range: 10.98–16.20). However, age was significantly associated with knowledge in the home and work environment (Chi^2^ = 19.27, *p* = 0.037, *V* = 0.093), with older participants demonstrating greater awareness. This is consistent with Dragano et al. (31), who found that younger workers have less access to occupational safety resources. The pattern of higher moderate perceived exposure among 17–24-year-olds across all domains is likely attributable to higher rates of processed food consumption, reliance on on-campus fast food, and limited formal environmental health training.

From an explicit gender perspective, a statistically significant association was identified between gender and self-reported perceived exposure in the home and work environments (Chi^2^ = 7.89, *p* = 0.019, *V* = 0.084), with male respondents reporting higher perceived exposure than female respondents. After multivariate adjustment (Table 4), female gender was independently associated with higher perceived exposure in the Food and Water domain (OR = 1.66, *p* = 0.004) and the Lifestyle domain (OR = 1.50, *p* = 0.013), while males showed higher perceived exposure in the Home and Work domain (OR for female = 0.68, *p* = 0.011). This three-way gender pattern indicates that behavioral exposure pathways vary by domain: males are more exposed through home and work environments, which is consistent with higher occupational and environmental exposures, whereas females may be more exposed through food and lifestyle-related behaviors (32). When it comes to the workplace, Scientific Colleges were independently associated with higher Food and Water perceived exposure compared to Literature Colleges (OR = 1.50, *p* = 0.028). This could indicate that science students engage more with processed and stored food products in laboratory and campus settings, or are more sensitive to reporting such exposures, given their scientific training.

Regarding governorate of residence, the descriptive chi-square analyses showed no significant association between governorate and self-reported perceived exposure levels across any domain (all *p* > 0.05). However, the governorate was significantly associated with food and water knowledge in Al-Ahmadi (Chi^2^ = 27.08, *p* = 0.003, *V* = 0.110), where proximity to the petroleum and industrial complex may heighten environmental health awareness. In contrast, multivariate logistic regression (Table 4) revealed that governorate was a significant independent predictor of Lifestyle perceived exposure, with residents of Al-Ahmadi (OR = 1.52, *p* = 0.034), Hawali (OR = 1.58, *p* = 0.045), and Mubarak Al-Kabeer (OR = 1.64, *p* = 0.030) showing higher odds than residents of Farwaniya (36). This finding suggests that, once other socio-demographic variables are controlled for, residential location does play a meaningful role in Lifestyle perceived exposure that was masked in the unadjusted Chi-square analyses. Future research should explore the specific lifestyle behaviors driving this governorate-level variation.

Administrative and faculty members showed descriptively higher self-reported perceived exposure compared to students across all three domains. However, after multivariate adjustment, occupation was not a significant independent predictor in any domain (Faculty OR range: 1.62–3.48, all *p* ≥ 0.05; Administrative OR range: 0.74–1.74, all *p* ≥ 0.05). This suggests that the apparent occupational differences in unadjusted analyses are confounded by other socio-demographic variables, particularly gender and workplace.

Female participants demonstrated more knowledge and awareness than their male counterparts. In the Food and Water domain, females displayed higher knowledge (32.8% high vs. 24.4% in males, Chi^2^ = 9.60, *p* = 0.008). Female participants were also 68% more concerned about outdoor air quality than males (33, 35). It should be noted that the smaller male subsample (*n* = 234) reduces statistical power in gender comparisons and warrants cautious interpretation.

Faculty members exhibited high knowledge across all three domains, suggesting their potential to disseminate information to students and administrative staff. Students from scientific colleges demonstrated a superior understanding of perceived exposure and preventive strategies, as science-related fields incorporate these topics into their curricula.

### 4.1 Multivariate findings

Binary logistic regression (Table 4) was used to identify which factors independently predicted whether participants reported moderate-to-high perceived exposure across the three domains. Several consistent patterns emerged. Gender stood out as one of the strongest and most reliable predictors — women were more likely than men to report higher perceived exposure in the Food & Water and Lifestyle domains, though interestingly, the opposite was true for the Health & Wellness domain. Age also played a role, with participants aged 32–39 being somewhat more likely to report higher Lifestyle exposure compared to the youngest group (17–24 years), with an odds ratio of 1.57 (*p* = 0.024). In terms of academic background, students from Scientific Colleges reported greater perceived exposure to Food and Water risks than their peers (OR = 1.50, *p* = 0.028). Residential location further shaped Lifestyle exposure perceptions, with participants from Al-Ahmadi, Hawali, and Mubarak Al-Kabeer showing notably different patterns compared to other governorates.

Taken together, gender and place of residence emerged as the most consistent correlates of perceived exposure, though the specific patterns varied depending on the domain in question. It should be noted, however, that effect sizes across the chi-square analyses were modest at best (Cramer’s V ≤ 0.13), and given the cross-sectional nature of the study, no causal conclusions can be drawn from these associations.

## 5 Limitations

The following limitations should be considered when interpreting the findings of this study:

1. Self-report bias: All exposure and knowledge data are based on self-report and do not reflect biomonitored or objectively measured toxin levels. Social desirability bias may lead to under-reporting of stigmatized exposures or over-reporting of preventive behaviours.
2. Student overrepresentation: Students constitute 92.5% of the sample, reflecting the university-based convenience sampling strategy. Findings may not generalize to the broader Kuwaiti adult population.
3. Gender imbalance: The sample is predominantly female (78.9%), broadly reflecting campus demographics but limiting statistical power in gender-comparative analyses.
4. Voluntary online participation bias: Self-selection may inflate knowledge scores among more environmentally aware participants.
5. Binary scoring conservatism: The binary scoring approach may underestimate cumulative perceived exposure burden for intermittent behaviours. Future studies should consider weighted scoring schemes.
6. Instrument validation: While internal consistency was acceptable-to-good (Cronbach’s alpha = 0.71–0.79), the scoring tool has not been externally validated against biomonitored exposure data.
7. Cross-sectional design: The design precludes causal inference. All associations are descriptive and hypothesis-generating.

## 6 Conclusion

This study identified moderate levels of self-reported perceived environmental toxin exposure among a large proportion of respondents at a Kuwaiti university campus, particularly through food and water pathways. Moderate perceived exposure was the most prevalent category across all three domains. Higher perceived exposure levels were more frequently reported among younger participants, despite older age groups demonstrating greater knowledge of environmental risks and preventive measures.

Multivariate logistic regression revealed that female gender was an independent predictor of perceived exposure across all three domains, with distinct directional effects: females reported higher Food and Water and Lifestyle perceived exposure but lower Home and Work perceived exposure compared to males. Governorate of residence was a significant independent predictor of Lifestyle perceived exposure, with Al-Ahmadi, Hawali, and Mubarak Al-Kabeer residents showing higher odds than Farwaniya residents. These governorate-level associations were not apparent in unadjusted chi-square analyses, highlighting the value of multivariate adjustment in revealing masked associations.

Knowledge levels were consistently higher among females, faculty members, and respondents affiliated with scientific colleges. Leveraging faculty expertise through campus-wide educational initiatives may represent an effective strategy to reduce environmental exposure risks and enhance preventive behaviours. Future interventional research is needed to establish whether targeted programs effectively reduce perceived or actual environmental toxin exposure in university settings. Limitations of this study are discussed fully in Section 5.

## Data Availability

The original contributions presented in the study are included in the article/supplementary material, further inquiries can be directed to the corresponding author.
